# Characterization of Lewy Body copathology in early AD clinical trial population demonstrates similarities and differences compared to natural history studies in Alzheimer's disease patients

**DOI:** 10.1002/alz70856_107102

**Published:** 2026-01-08

**Authors:** David Verbel, Hongmei Niu, Larisa Reyderman, Michael C. Irizarry, Pallavi Sachdev

**Affiliations:** ^1^ Eisai Inc., Nutley, NJ, USA

## Abstract

**Background:**

Existing copathologies, such as Lewy body (LB) disease, can introduce heterogeneity to AD pathogenesis and presentation of symptoms and likely influence disease progression. A seed amplification assay (SAA) that detects aggregated, misfolded α‐synuclein (α‐syn) can detect LB. Incorporation of SAA can characterize disease heterogeneity in AD and potentially predict disease progression.

**Methods:**

A validated SAA was used to characterize α‐syn SAA status (Amprion Clinical Laboratory) in baseline CSF samples collected from a Phase 3 program for elenbecestat in subjects with MCI or mild dementia due to AD (NCT02956486). Samples were selected by PET visual read status. The sample set was enriched using subjects considered to be progressors or non‐progressors based on change in cognition (CDR‐SB) at 18 months, before assessment of SAA status. Results of α‐syn status were reported as Detected, Not Detected, or Indeterminate. The proportion of samples defined as Detected and Not Detected were summarized by amyloid status. Cognition, at baseline and longitudinally, was summarized by amyloid and α‐syn status. Available baseline A/T/N biomarkers were also summarized and compared.

**Results:**

Among the 201 samples that were included in the analyses, 15% of amyloid+ and 8% of amyloid‐ MCI/early AD subjects were determined to be SAA+ and hence determined to have LB copathology. In comparison, in ADNI, 17% of cognitively unimpaired (CU) subjects; 20% of MCI subjects and 39% of AD subjects had synuclein copathology (Tosun et al Alzheimer's Dement. 2024). In BioFinder2, synuclein copathology was detected in 8%, 17% and 23% of CU, MCI, and AD subjects, respectively (Palmqvist et al and Quadalti et al Nature Medicine 2023). The longitudinal trajectory of amyloid positive subjects with α‐syn copathology seemed to have faster cognitive decline in natural history studies. In the Phase 3 samples, the longitudinal cognitive trajectory showed trend towards faster decline over 18 months in the amyloid+ subjects with α‐syn copathology; however, this did not appear significant.

**Conclusion:**

LB, a common copathology of AD, may be a source of heterogeneity. Incorporation of SAA, a specific biomarker of LB‐pathology, can help account for disease heterogeneity and potentially improve assessment of treatment response in amyloid and synuclein status confirmed AD target population.

